# Supplementary material for: A high-resolution mRNA expression time course of embryonic development in zebrafish
Source: eLife. 2017 Nov 16;6:e30860. doi: 10.7554/eLife.30860 (PMC5690287; doi:10.7554/eLife.30860)
Supplement: Supplementary file 6. [file elife-30860-supp6.zip › biolayout-clusters-files/Cluster184-genes.html]

Cluster184


# Cluster184: Genes

| Ensembl ID | Gene Name | Chr | Start | End | Biotype |
| --- | --- | --- | --- | --- | --- |
| ENSDARG00000102545 | BX537137.2 | 4 | 36423137 | 36428737 | protein\_coding |
| ENSDARG00000079328 | ENSDARG00000079328 | 22 | 5674514 | 5694306 | protein\_coding |
| ENSDARG00000035719 | arl5c | 3 | 15984576 | 15992257 | protein\_coding |
| ENSDARG00000057323 | e2f8 | 7 | 16324269 | 16346257 | protein\_coding |
| ENSDARG00000007387 | irf2a | 1 | 39141709 | 39162116 | protein\_coding |
| ENSDARG00000071790 | zgc:171318 | 22 | 1894395 | 2091341 | protein\_coding |
| ENSDARG00000096012 | znf1115.1 | 4 | 44457514 | 44475284 | protein\_coding |
| ENSDARG00000093831 | znf1121 | 4 | 29893649 | 29900097 | protein\_coding |
